# Menthol Smoking and Nicotine Dependence among Black/African American Women Smokers Living in Low-Resource, Rural Communities

**DOI:** 10.3390/ijerph182010877

**Published:** 2021-10-16

**Authors:** Dina M. Jones, Margarete C. Kulik, Lourdes Baezconde-Garbanati, Sandilyn Bullock, Mignonne C. Guy, Pebbles Fagan

**Affiliations:** 1 Center for the Study of Tobacco, Department of Health Behavior and Health Education, Fay W. Boozman College of Public Health, University of Arkansas for Medical Sciences, 4301 West Markham Street, Box #820, Little Rock, AR 72205, USA; maggie.kulik@ucop.edu (M.C.K.); sbbullock@uams.edu (S.B.); pfagan@uams.edu (P.F.); 2 Tobacco-Related Disease Research Program, Research Grants Program Office, University of California, Office of the President, 1111 Franklin Street, 11th Floor, Oakland, CA 94607, USA; 3 Institute for Health Promotion and Disease Prevention, Department of Population and Public Health Sciences, Keck School of Medicine, University of Southern California, SSB 302M 2001 N. Soto Street, Los Angeles, CA 90033, USA; baezcond@usc.edu; 4 Department of African American Studies, College of Humanities and Sciences, Virginia Commonwealth University, 816 W. Franklin Street, Room 201, Richmond, VA 23284, USA; mguy@vcu.edu

**Keywords:** menthol, nicotine dependence, tobacco, African Americans, rural, low income, cigarette, little cigar, cigarillo, cessation

## Abstract

Black/African American women from low-resource, rural communities bear a disproportionate burden of tobacco-related morbidity and mortality. This study examined associations between menthol smoking and socioeconomic deprivation with nicotine dependence and quitting behaviors among Black/African American women cigarette and/or little cigar/cigarillo smokers, aged 18–50 living in low-resource, rural communities. Baseline survey data from a randomized controlled behavioral/intervention trial (#NCT03476837) were analyzed (*n* = 146). Outcomes included time to first tobacco product (cigarette/little cigar/cigarillo) use within 5 min of waking, Fagerstrom Test for Nicotine Dependence (FTND) score, and ever attempting to quit cigarettes. Socioeconomic deprivation measures included education, income, and receiving supplemental nutritional assistance (SNAP) program benefits. In adjusted regression analyses, menthol smoking was associated with both greater FTND scores and time to first tobacco product use within 5 min of waking, but not ever attempting to quit cigarettes. Regardless of menthol status, only 25.0% of smokers reported that they would quit smoking if menthol cigarettes were banned. The proportion of smokers who smoked their first tobacco product within 5 min of waking increased slightly with greater socioeconomic deprivation. Additional research and targeted efforts are needed to reduce nicotine dependence among Black/African American women smokers living in rural, low-resource communities where access to cessation services is limited.

## 1. Introduction

Black/African American women from the Southern United States (U.S.) continue to disproportionately suffer from tobacco-related health consequences, including lung cancer. For example, since 2002, lung cancer incidence rates have declined among Black/African American women in the U.S. In Arkansas, however, these rates have remained stable and are higher among Black/African American women [1,2]. Differences in national and regional lung cancer incidence rates among Black/African American women may be attributed to differences in smoking.

Recent prevalence estimates of current cigarette smoking are 13% among Black/African American women in the U.S. compared to 19% of Black/African American women in Arkansas [3,4]. Arkansas, classified as a “Tobacco Nation” state, is one of 13 states with high smoking rates, consistently poor implementation of tobacco prevention and control policies, and lacks an adequate health care infrastructure [5]. Black/African American women who live in Tobacco Nation states like Arkansas are among those with the lowest socioeconomic status in the nation, are more likely to have low levels of education, and are more likely to be under/unemployed compared to women who do not live in a Tobacco Nation state [5,6]. Indicators of socioeconomic deprivation such as poverty, low levels of education, and receipt of supplemental nutritional assistance program (SNAP) benefits are associated with a higher prevalence of smoking in general [7,8] and excess lung cancer mortality among females [9] and may contribute to the higher rates of smoking and related lung cancer incidence rates among Black/African American women in Arkansas.

In addition to higher rates of smoking, socioeconomically deprived women are more likely than more advantaged women to smoke menthol cigarettes [10], have higher levels of nicotine dependence, and have greater difficulty quitting smoking [11,12]. Menthol is a flavoring additive commonly used in tobacco and has cooling, anesthetic, and analgesic properties that promote smoking maintenance and nicotine dependence [13,14]. Menthol is used in nearly all tobacco products, including hookah, smokeless tobacco, e-cigarettes and other electronic nicotine delivery systems (ENDS), cigars, and cigarettes [15]. As a characterizing flavor, however, the most widely sold menthol tobacco products are cigarettes, large cigars, and little cigars and cigarillos (LCCs) [16]. Moreover, menthol-flavored cigarettes are disproportionately used among Black/African American women smokers and are commonly used among rural, Southern, less educated, and low-income smokers.

Although nearly 70–90% of all Black/African American smokers use cigarettes characterized as menthol-flavored, menthol smoking is more common among Black/African American women than men [10], and in 2019, the prevalence of menthol cigarette smoking among past 30-day Black/African American women smokers was 84.9% [11]. Moreover, Black/African American women smokers who resided in non-metropolitan (vs. large metropolitan) counties had a higher prevalence of menthol smoking (91.8% vs. 84.4%) [11]. Rates of menthol smoking have also remained stable from 2005–2015 and are highest among Blacks/African Americans (11.9%) and those residing in the U.S. South (5.2%), with a less than high school education (6.6%), and with a family income less than $35,000 (7.0%) [17]. This is especially concerning, given the increased cessation difficulties associated with menthol smoking.

Research indicates that menthol cigarettes are more addictive than non-menthol cigarettes, and they increase difficulty in quitting among Black/African Americans and women [13,18]. For example, prior studies indicate that women who smoke menthol cigarettes have greater nicotine dependence than non-menthol smokers [19,20]. Studies also show that menthol cigarette and low-income smokers, especially those who are Black/African American, are more likely to smoke LCCs [21,22,23,24,25], which is problematic because LCCs carry the same health risks as cigarettes and can deliver enough nicotine to maintain dependence [26,27]. Additionally, a disproportionate burden of socioeconomic deprivation combined with menthol smoking may further increase nicotine dependence and smoking cessation difficulty among Black/African American women smokers [5,6]. As such, there is a critical need to understand nicotine dependence and other factors that impede smoking cessation among Black/African American women smokers, especially those from low-resource, rural, and/or Southern U.S. communities.

However, there is a significant gap in knowledge on how both menthol and the experience of socioeconomic deprivation influence nicotine dependence among Black/African American women smokers from low-resource, rural communities. Because the proportion of Black/African American women who smoke non-menthol cigarettes is relatively low [10], it is often difficult to assess how menthol influences nicotine dependence among these women. Thus, the purpose of this study was to examine the association between (1) menthol smoking with nicotine dependence and quitting behaviors and (2) socioeconomic deprivation and nicotine dependence among Black/African American women cigarette and/or little cigar/cigarillo smokers, aged 18–50, who live in low-resource, rural communities. Examining these relationships will help us better understand the mechanisms that drive tobacco-related disparities among Black/African American women who live in low-resource and rural communities, like those in Arkansas, and inform future interventions to help them successfully quit.

## 2. Materials and Methods

### 2.1. Study Setting

In 2019, the Families Rising to Enforce Smokefree Homes (FRESH) study began recruiting Black/African American women caregivers who are current cigarette and/or little cigar and cigarillo (LCC) smokers and reside in Lee and Philips counties in rural Arkansas. The FRESH study is an ongoing, randomized controlled behavioral intervention trial that aims to increase comprehensive smokefree policies in the home as a primary outcome and quitting smoking as a secondary outcome (clinical trial #NCT03476837) among those eligible in the intervention counties. The present study was designed to be representative of Black/African American women caregivers who currently smoke cigarettes and/or LCCs and reside in rural, low-resource communities in Arkansas.

As such, our sample was recruited from Lee and Phillips counties, located in the Delta region of Arkansas, where smoking prevalence is approximately 29% and 27%, respectively, and the population count is less than 20,000 residents in each county [28]. The social, economic, and political conditions in the two counties are similar to those observed in other Tobacco Nation states [5], and residents in Lee and Phillips counties have similar sociodemographic and socioeconomic backgrounds [28]. For example, residents in Lee and Phillips counties have among the lowest life expectancies in the nation, and more than 50% of residents in both counties are Black/African American [28,29,30]. Although more than 40% of the residents in both counties are Medicaid eligible, contributors to the low life expectancies in both counties include high rates of poverty (~35%) and food insecurity (>20%) [28,29,30,31]. Few hospitals or state or federally qualified health clinics are located in either county [28]. Moreover, the ratio of primary care providers per patients is 3340:1 in Lee and 2901:1 in Phillips County and the nearest medical centers are more than 20 miles away from both counties [28].

### 2.2. Sampling, Eligibility, and Recruitment

Those eligible to participate in the study (1) were women who self-identified as Black/African American; (2) were aged 18–50; (3) currently resided in the intervention counties (i.e., Lee or Phillips counties, AR); (4) spoke English; (5) provided written informed consent; (6) had a working phone, home address, and email; (7) were the primary caregiver (birth parent, guardian) of at least one child, aged 6 months to 14 years old; (8) were the primary decision maker in the home; (9) smoked cigarettes and/or LCCs for at least 1 year and in the past 30 days; and (10) were of low income, as defined by any indicator (e.g., Medicaid; Earned Income Tax Credit; Children’s Health Insurance Plan (ARKids); subsidized housing; child care subsidies; food stamps/SNAP benefits; low-income energy assistance; free/reduced lunch program; Head Start program).

**Figure 1:**
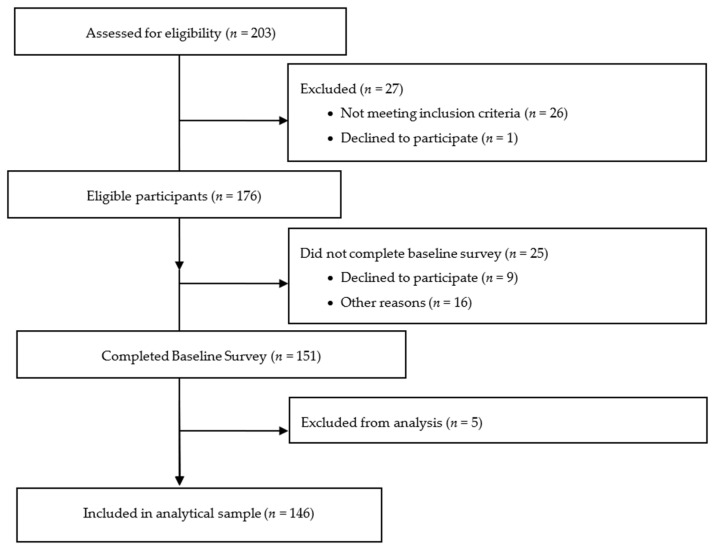
Diagram showing the number of participants screened for eligibility, determined to be eligible, and enrolled as of 26 July 2021.

The present study utilized non-probability sampling methods, including homogenous convenience sampling and peer referrals [32,33]. Non-probability sampling methods are acceptable and commonly used in community-based studies to recruit underrepresented, hard-to-reach populations, such as Black/African American women smokers from low-resource, rural communities, given that fully enumerated sampling frames are not typically or easily made available [32,34,35]. Research staff used word-of-mouth; radio, newspaper, and social media advertisements; flyers; in-person and socially-distanced community events (i.e., recruitment events with housing authorities, community centers, and food distribution events with local food banks); and provided enrolled participants and community contacts with incentives for referrals to recruit participants into the study. Trained research staff screened potential participants by telephone or in-person. Potential participants were emailed the consent form and consented to being in the study prior to completing the baseline survey online. A flow chart depicting participant screening, eligibility, and enrollment as of 26 July 2021 is depicted in Figure 1.

### 2.3. Procedures for Survey Administration

Baseline surveys were collected prior to randomization. Trained research staff administered baseline surveys in the home or at an agreed upon location, using computer-based surveys (iPad or laptop) or using online self-administered surveys. Staff were available by phone or email to answer participant questions about the consent form or baseline survey. All participants received a $30 reloadable research gift card (i.e., ClinCard) and a one-page fact sheet about quitting smoking at the end of the visit.

### 2.4. Measures

The baseline survey among cigarette and little cigar/cigarillos smokers measured sociodemographic and tobacco user characteristics.

#### 2.4.1. Sociodemographic Characteristics

Data were collected on age (calculated from participant date of birth), race (Black/African American only, Biracial/multiracial with Black/African American), sexual orientation and gender identity (heterosexual/straight, homosexual/gay/lesbian, bisexual, transgender, other, or not sure), marital status (married, widowed, divorced, separated, never married, living with partner), employment status (full-time, part-time, not currently working for pay), insurance status (insured, uninsured), and body mass index (BMI). Hispanic ethnicity was collected but not reported, due to insufficient sample size. Additional sociodemographic characteristics, including educational attainment, annual household income, and receipt of SNAP benefits in the past 12 months, were used to create a measure of socioeconomic deprivation and are described in the below section.

#### 2.4.2. Tobacco Use Characteristics

##### Menthol Cigarette Smoking Status

Participants who indicated that they had smoked at least 100 cigarettes in their lifetime were asked: “Do you currently smoke cigarettes every day, some days, or not at all?” Those who responded “every day” or “some days” were considered current cigarette smokers. Current cigarette smokers were asked about the usual type of cigarette they smoked (menthol, non-menthol, or no usual type) and the following question, “When you smoked cigarettes during the past 30 days (month), did you smoke…?”, with response options including only menthol, mostly menthol, half and half, mostly non-menthol, and only non-menthol. Those who reported smoking only menthol cigarettes in the past 30 days were considered current menthol cigarette smokers.

##### Cigarette Smoker Characteristics

Current cigarette smokers were asked about the age at which they first smoked cigarettes fairly regularly, the number of cigarettes they smoked per day in the past 30 days, the number of days that they smoked cigarettes in the past 30 days, and what they would be most likely to do if menthol cigarettes were no longer sold (e.g., switch to a non-menthol cigarette, switch to LCC smoking, switch to large/premium cigars, switch to electronic cigarettes, switch to smokeless tobacco, switch to a pipe, switch to IQOS, switch to hookah, quit any cigarette smoking, switch to marijuana, none of the above, and I don’t smoke menthols).

##### Menthol LCC Smoking Status

Participants who indicated that they had ever smoked, even one or two puffs, a filtered little cigar or cigarillo were asked, “Do you now use little cigars or cigarillos every day, some days, or not at all?” Those who reported using LCCs every day or some days were considered current LCC smokers. Current LCC smokers were asked what flavor of LCC they usually smoke (e.g., menthol or mint, clove, spice or nut, tobacco, fruit, chocolate, vanilla or cream, an alcoholic drink, beverages, coffee/tea, candy, desserts, other sweet flavor, some other flavor, I don’t use a flavor, and don’t know) and “During the past 30 days, was the little cigar/cigarillo that you smoked…” with the same response options.

##### LCC Smoker Characteristics

Current LCC smokers were asked about the age at which they first smoked LCCs fairly regularly, the number of LCCs they smoked per day in the past 30 days, the number of days that they smoked LCCs in the past 30 days, the brand of LCCs usually smoked in the past 30 days (Black & Mild, Swisher Sweet, Phillies, White Owl, Garcia y Vega, Al Capone, Captain Black, Game, Backwoods, Dutch Master, Prime Time, and Other), and current (every day or some day) use of cigars/LCCs as blunts.

##### Composite of Any Current/Past 30-Day Menthol Smoking Status

Given the high rates of usual menthol smoking (see Results section), we created a more conservative, composite measure of current menthol smoking status by combining past 30-day (only) menthol cigarette smoking and past 30-day menthol LCC use. Participants were categorized as any menthol or non-menthol smokers.

#### 2.4.3. Other Substance Use Characteristics

Participants also reported current (every day or some day) use of other tobacco products (premium/large cigars, hookah, pipe tobacco, IQOS/heated tobacco, and smokeless tobacco), alcohol, marijuana, and other illicit drugs (cocaine, heroin, opioids, fentanyl, ecstasy, methamphetamines, bath salts, or K2).

#### 2.4.4. Socioeconomic Deprivation Measures

Several variables were used to measure the depth of poverty/socioeconomic deprivation. We assessed level of educational attainment (assessed using a 13-level response option ranging from ‘No schooling completed’ to ‘Doctorate degree (for example PhD, EdD)), annual household income (assessed using a 12-level response option ranging from ‘less than $10,000 per year to $150,000 or more), and whether participants received SNAP benefits in the past 12 months (yes vs. no). Level of educational attainment was categorized as ‘less than high school’, ‘high school diploma or Graduate Equivalency Degree (GED)’, and ‘some college, associate’s degree, bachelor’s degree or higher’ in bivariate and regression analyses and dichotomized to ‘12th grade education, no diploma’ or lower vs. high school diploma or greater in social deprivation analyses. Annual household income was dichotomized to <$10,000 vs. ≥$10,000 in all analyses.

#### 2.4.5. Outcome Variables

##### Nicotine Dependence

We examined nicotine dependence using the Fagerstrom Test of Nicotine Dependence (FTND) [36] and time to first tobacco product use [37]. The FTND is a validated scale consisting of 7 items used to assess nicotine dependence among cigarette smokers [36]. Current cigarette and LCC smokers also reported their time to first cigarette/LCC (i.e., how soon they smoked their first cigarette after waking (within 5 min, 6–30 min, 31–60 min, or after 60 min)). Time to first product use is often used as a stand-alone measure of nicotine dependence (use within first ≤5 min of waking vs. >5 min of waking). Time to first cigarette and/or LCC use measures were combined for a composite time to first tobacco product use measure.

##### Quitting Behaviors

Current cigarette smokers were asked whether they had ever tried to quit smoking cigarettes and whether they were planning to quit cigarette smoking within the next 30 days (yes, no, don’t know). Among those who had ever tried to quit cigarette smoking, we assessed whether they tried to quit cigarette smoking in the past year (yes vs. no).

### 2.5. Sample and Analysis

A total of 151 participants completed the baseline study survey as of 26 July 2021. Five participants were excluded from the analytic sample because their cigarette and LCC smoking statuses could not be assessed. Thus, the current analytic sample included 146 participants (Figure 1). Analyses were conducted in SAS 9.4 (Cary, NC, USA) and Stata 15.1 (StataCorp, College Station, TX, USA), and we conducted univariate and bivariate analyses (Wald χ^2^, Fishers exact, t, and Mann Whitney U tests) to compare the sociodemographic, tobacco use, and other substance use characteristics by any menthol smoking status.

Multivariable logistic regression and 95% confidence intervals (CIs) were used to examine the associations between (1) any menthol smoking status and the time to first tobacco product use within 5 min of waking (yes vs. no) and (2) menthol smoking status and ever attempting to quit cigarettes (yes vs. no). Multivariable linear regression models were used to examine the association between menthol smoking status and FTND score. Model 1 adjusted for age, income, education, sexual orientation, and marital status (dichotomized as married vs. all else/not married in all regression analyses). In analyses with the outcomes of time to first tobacco product use and ever attempting to quit cigarettes, model 2 adjusted for age, income, education, sexual orientation, marital status, other tobacco product use, log transformed number of cigarettes smoked per day, and number of days one had smoked cigarettes in the past 30 days. In analyses with the outcome of FTND score, model 2 adjusted for age, education, income, sexual orientation, marital status, other tobacco product use, and the number of days participants smoked cigarettes in the past 30 days. For all analyses, a *p*-value < 0.05 was considered statistically significant.

We constructed a measure of socioeconomic deprivation aimed to examine whether nicotine dependence varied with increasing exposure to multiple socioeconomic deprivation indicators, adapting an approach used by Barbeau and colleagues [8]. In socioeconomic deprivation analyses, low education was defined as an educational attainment of “12th grade, no diploma” or lower, and low income was considered to be an annual household income <$10,000 a year. To conduct these analyses, we first examined the proportion of smokers who reported using their first tobacco product (cigarette or LCC) within 5 min of waking among those with low education (i.e., the lowest level of socioeconomic deprivation). Next, we examined the proportion of smokers who reported using their first tobacco product within 5 min of waking among those who experienced both low education and a low household income. Finally, we examined the proportion of smokers who used their first tobacco product within 5 min of waking among those who experienced low education, low income, and received SNAP benefits (i.e., the deepest level of socioeconomic deprivation). We also examined the time to first cigarette use and FTND score for each level of socioeconomic deprivation among cigarette smokers only.

## 3. Results

### 3.1. Menthol Cigarette Use among Black/African American Women Who Currently Smoke Cigarettes and/or LCCs

Among current smokers (*n* = 120), 91.6% reported usually smoking menthol cigarettes, and among current LCC smokers (*n* = 59), 25.9% reported usually smoking menthol-flavored LCCs. Rates of past 30-day menthol smoking were lower as 82.5% of cigarette smokers reported only smoking menthol cigarettes, and 18.6% of LCC smokers reported smoking menthol LCCs in the past 30 days. Overall, 69.2% (*n* = 101) of participants reported using any menthol product in the past 30 days, and this composite measure of any current menthol smoking in the past 30 days was used for the analyses below. Among past 30-day menthol only cigarette smokers, 9.1% (*n* = 9) also reported smoking menthol LCCs in the past 30 days (data not shown). Among past 30-day menthol LCC smokers, 81.8% (*n* = 9) reported only smoking menthol cigarettes in the past 30 days (data not shown).

### 3.2. Sociodemographic Characteristics of Black/African American Women Smokers by Menthol Smoking Status

Sociodemographic characteristics by any current menthol smoking status are presented in Table 1. On average, participants were 33.3 years old, and over one-quarter identified as a sexual minority. Most women were never married, had a high school diploma or GED, had an annual household income of less than $10,000, and received SNAP benefits in the past year. Menthol smokers were significantly more likely than non-menthol smokers to have less than a high school education. We did not observe any other differences in sociodemographic characteristics by menthol smoking status. Although not statistically significantly different, compared to non-menthol smokers, menthol smokers were generally older and more likely to be married (Table 1).

### 3.3. Tobacco and Other Substance Use Characteristics

Smoker and other substance use characteristics by any current menthol smoking status are presented in Table 2. Overall, most participants smoked cigarettes daily, smoked cigarettes on an average of 20.2 days in the past 30 days, and smoked a median number of 8.0 cigarettes per day. Nearly one-third of participants also reported currently using a non-cigarette or non-LCC tobacco products (e.g., premium/large cigars, hookah, pipe tobacco, IQOS/heated tobacco, or smokeless tobacco). When asked what they would do if menthol cigarettes were banned, 25.0% of cigarette smokers reported that they would quit any cigarette smoking, and 18.1% reported that they would switch to a non-menthol cigarette. Half reported a time to first tobacco product use of within 5 min of waking, and 76.7% reported smoking their first cigarette within 30 min of waking (data not shown). Two-thirds of cigarette smokers had an FTND score of ≥4 (data not shown), and the average FTND score among cigarette smokers was 4.5. Among cigarette smokers, over half had ever tried to quit smoking cigarettes, only half of whom made a past-year cigarette quit attempt (Table 2).

Compared to non-menthol smokers, menthol smokers were significantly more likely to smoke cigarettes daily, smoke cigarettes on a greater number of days in the past 30 days, smoke a greater number of cigarettes per day, and smoke any product within 5 min of waking. Current menthol smokers were significantly less likely than non-menthol smokers to report current other tobacco product use. Current menthol smokers also had significantly greater FTND scores, on average, than non-menthol smokers. Although not statistically significantly different, menthol smokers were generally less likely to have ever tried to quit smoking cigarettes than non-menthol smokers (Table 2).

### 3.4. Multivariable Linear and Logistic Regression Predicting Time to First Tobacco Product Use, FTND Score, and Ever Attempting to Quit Cigarettes from Menthol Smoking Status

Multivariable linear and logistic regression results are presented in Table 3. Model 1 showed that following adjustment for sociodemographic characteristics, any current menthol smoking was associated with higher odds of the time to first tobacco product use being within the first 5 min of waking (AOR: 4.10 [95% CI: 1.73, 9.70]) and higher FTND scores among menthol cigarette smokers (B = 1.49 [SE: 0.56]). The significant associations between any menthol smoking and time to first tobacco product use (AOR: 5.90 [95% CI: 1.16, 22.33] and FTND score (B = 1.16 [SE: 0.58]) persisted following adjustment for sociodemographic characteristics and smoker characteristics. No association was found between menthol smoking status and ever attempting to quit cigarettes (Table 3).

### 3.5. Socioeconomic Deprivation by Time to First Tobacco Product Use, Time to First Cigarette Use, and FTND Score

Table 4 shows nicotine dependence at different levels of depth of socioeconomic deprivation for all smokers. Due to the small sample size of non-menthol smokers, we did not stratify these analyses by menthol smoking status. Among the entire sample, nicotine dependence increased with the depth of socioeconomic deprivation. Overall, 47.9% of participants smoked a cigarette and/or LCC within 5 min of waking. The percentage of participants who smoked their first tobacco product within 5 min of waking increased to 59.0% among those with the lowest level of socioeconomic deprivation (i.e., low education) and 65.2% among those with the highest level of socioeconomic deprivation (i.e., low education, low income, and receipt of SNAP benefits).

Among cigarette smokers, nicotine dependence also slightly increased with the depth of socioeconomic deprivation. Overall, 50.0% of cigarette smokers indicated that they smoked their first cigarette within 5 min of waking. The percentage of cigarette smokers who smoked their first cigarette within 5 min of waking increased to 59.5% among those with the lowest level of socioeconomic deprivation and 63.6% among those with the highest level of socioeconomic deprivation. No difference was found in FTND score by depth of socioeconomic deprivation (Table 4).

## 4. Discussion

In sum, our study found that current menthol smoking was associated with two separate indicators of nicotine dependence among Black/African American women smokers who reside in rural, low-resource communities. Compared to non-menthol smokers, menthol smokers had significantly greater FTND scores and higher odds of smoking their first tobacco product within 5 min of waking, following adjustment for sociodemographic and tobacco use characteristics. No association was found with ever attempting to quit cigarettes and menthol smoking in adjusted analyses. However, we found that the likelihood of being nicotine dependent slightly increased with greater exposure to socioeconomic deprivation.

In the present study, Black/African American women menthol smokers had moderate levels of nicotine dependence, and in adjusted analyses, menthol smoking status was associated with greater nicotine dependence, using two different measures. Few studies have examined menthol smoking and FTND, and results of prior studies on menthol smoking and nicotine dependence vary depending on the measure used to assess nicotine dependence. For example, in a review of studies on menthol cigarette smoking and nicotine addiction, five out of six studies that measured nicotine dependence using time to first cigarette use found a positive association with menthol smoking [38]. Conversely, all studies that measured nicotine dependence using FTND (*n* = 3) found no association with menthol smoking. [38] Thus, our results related to menthol smoking and FTND are especially novel considering that we observed a significant association. Interestingly, the FTND score of menthol smokers in the present study (mean 4.7 and 66% of women with a score ≥4) was the exact same as Black/African American adult/women menthol smokers in two of the studies [39,40] from the review. However, average FTND score in our study was higher than one of the studies, which was conducted among a diverse (46.9% Black/African American) sample of adolescents [41]. Separately, Smith and colleagues found that FTND scores were higher among Black and White menthol smokers compared to non-menthol smokers, but their sample was predominantly white (70.2% of menthol and 100% of non-menthol smokers) [42]. Further studies are needed to better understand the impact of menthol smoking status on FTND scores among Black/African American smokers.

Meanwhile, our findings related to menthol smoking status and time to first tobacco product/cigarette use are generally consistent with the available literature [38,39,40,41,42,43,44]; however, stratification by both race and gender are rare. For example, Muscat and colleagues found that only Black/African American menthol cigarette smokers were more likely (61% vs. 44%) to smoke their first cigarette within 15 min of waking, compared to non-menthol smokers [14]. The association remained significant among those who reported smoking 5–10 and 11–20 cigarettes per day [14], which is consistent with the present study, as participants were light smokers who smoked a median number of eight cigarettes per day. Conversely, Fagan and colleagues found that Black/African Americans who usually smoked menthol cigarettes had a longer time (20.9 min vs. 19.4 min) to their first cigarette compared to non-menthol smokers [43]. Importantly, neither Muscat and colleagues’ nor Fagan and colleagues’ studies stratified analyses by gender or measured nicotine dependence using the full FTND.

Analyses in St. Helen and colleagues’ cessation trial of Black/African American light smokers were stratified by gender, and both FTND and time to first cigarette use were examined [44]. However, nicotine dependence was greater among women smokers in the present study, despite our use of more stringent cutoffs for FTND (67.7% vs. 41.8% had an FTND score ≥ 4) and time to first cigarette use (50% smoked within 5 min of waking, and 76.7% vs. 71.4% smoked within 30 min of waking). Notably, compared to women in the present study, Black/African American women smokers in St. Helen and colleagues’ study were older and less socioeconomically deprived in terms of education (13.2% vs. 26.9% with less than high school education) and income (41.8% with an annual income ≥$21,600 vs. 34.5% with an annual income ≥$10,000) [44]. Future studies should seek to further clarify the extent to which menthol smoking contributes to a short time to first cigarette use among Black/African American women menthol smokers, especially those who are socioeconomically deprived and reside in low-resource, rural communities.

Although we found that menthol smoking status was associated with greater signs of dependence, menthol smoking status was surprisingly not associated with ever trying to quit smoking cigarettes. Prior studies have found that menthol smoking is associated with greater quitting difficulty among Black/African American menthol smokers, overall and by sex. For example, in a recent meta-analysis on menthol cigarette use and smoking cessation, nine studies were conducted among Black/African American smokers. In combined analyses of these studies, menthol smoking was associated with lower odds of smoking cessation (OR: 0.88 [95% CI: 0.78, 1.00]). Similarly, in Smith and colleagues’ community-based trial, menthol (vs. non-menthol) smokers had significantly fewer prior cigarette quit attempts at baseline, both overall and among Black/African American (vs. White) menthol cigarette smokers specifically [42]. Additionally, an interaction by race and sex was found among menthol smokers in longitudinal analyses, wherein Black/African American (vs. White) female menthol smokers had significantly lower cessation outcomes [42]. Thus, it is possible that our lack of observed findings regarding menthol smoking status and quitting behaviors may be due to a lack of statistical power/small sample size, rather than a lack of association. Additional research among Black/African American women and socioeconomically disadvantaged smokers is needed to assess the relationship between menthol smoking and quitting behaviors.

Our social deprivation findings are a novel contribution to the literature, and we found that the likelihood of being nicotine dependent increased slightly with increasing socioeconomic deprivation, as measured by educational attainment, annual household income, and receipt of SNAP benefits. Prior studies have linked nicotine dependence with individual measures of socioeconomic status. For instance, samples of predominantly White smokers have found that having a low education or low income is associated with greater nicotine dependence, as measured by the time to first cigarette use (≤5 min) [43], FTND [45], and heaviness of smoking index [46]. Similarly, in Branstetter and colleagues’ study, income was the only significant predictor of a time to first cigarette of within 5 min of waking among Black/African American participants [47]. Research has also linked food insecurity and deprivation with greater nicotine dependence [48] and showed that households that receive SNAP benefits are more likely to have at least one cigarette smoker [49]. However, very few studies have utilized composite measures to assess the impact of multiple forms of socioeconomic deprivation on smoking behaviors, especially nicotine dependence, both overall and among Black/African American women.

Barbeau and colleagues’ study, which guided our social deprivation analyses, found that cigarette smoking prevalence increased with increasing socioeconomic deprivation among Black women, but they did not examine nicotine dependence [8]. Meanwhile, a separate study found a higher predicted probability (40.9% vs. 30.3%) of time to first cigarette use within 30 min of waking among those with the lowest compared to the highest level of socioeconomic deprivation (i.e., low education/income vs. high education/income) [50]. However, this study was conducted among Norwegian smokers [50]. Nevertheless, the novel socioeconomic deprivation findings from the present study suggest that Black/African American women smokers, especially those from low-resource communities, may need more intensive support to eliminate their nicotine dependence and increase their likelihood of making a quit attempt and successfully quitting. Moreover, future interventions aimed at increasing successful smoking cessation among Black/African American women smokers from low-resource, rural communities may benefit by addressing, and if possible improving, the social and socioeconomic conditions of participants.

Our findings regarding menthol smoking status and sociodemographic and smoker characteristics are similar to findings from larger or national studies. Prior studies have found that menthol smoking and flavored LCC use is most prevalent among African Americans, those with less education, and those living in poverty [51,52,53]. Mattingly and colleagues found that from 2005–2015, menthol cigarette smoking remained stable among those aged 25–34, with less than a high school education, with a household income less than $35,000, and those residing in the South, but it decreased among females and non-Hispanic Blacks [17]. Despite these trends, the prevalence of menthol cigarette smoking was highest among smokers with these sociodemographic characteristics [17]. Interestingly, nearly all menthol LCC smokers in our study reported current menthol cigarette smoking, and menthol smokers were also more likely to smoke cigarettes daily. Our findings are supported by those from our prior study which found that African Americans who smoked menthol cigarettes daily (vs. occasional non-menthol smokers) had seven-times higher odds of flavored LCC use [21]. Although the present study is not generalizable to the overall population of Black/African American women smokers, it is not surprising that we found high rates of menthol smoking, given that our sample was recruited from rural, low-resource communities in the South and that the average participant age was 33 years old. When possible, future studies on Black/African American women menthol smokers should seek to recruit participants with diverse sociodemographic backgrounds and patterns of LCC use to better understand within group differences in the prevalence of menthol smoking.

The U.S. Food and Drug Administration (FDA) recently announced its commitment to issue proposed product standards within the next year to ban menthol as a characterizing flavor in cigarettes and ban all characterizing flavors, including menthol, in cigars [54]. However, it is not clear when a final rule will be implemented. Therefore, it is critically important to continue to identify effective strategies to help Black/African American women residing in low-resource, rural communities quit combustible tobacco successfully. In the present study, half of cigarette smokers had ever tried to quit smoking cigarettes, and less than 20% of the sample planned to quit smoking in the next 30 days. Moreover, we found that merely 25.0% reported that they would quit cigarette smoking if menthol cigarettes were no longer sold. A recent scoping review only found 11 papers that examined the effects of a hypothetical menthol ban in the U.S., eight of which used surveys [55]. The proportion of menthol smokers who reported that they would quit following a menthol ban in the present study was lower than most estimates from the review (25.3% vs. ≥35% to 65.9%) [55]. Our estimate was also lower (25% vs. 47%) than prior estimates among Black/African American menthol smokers [56,57]. However, our findings are consistent with three national studies of menthol smokers [57,58,59], although it is probable that our low estimate of quitting smoking following a hypothetical menthol ban was influenced by the rural and low resource background of participants. As the FDA considers how to best implement the ban on menthol cigarettes and characterizing flavors in cigars, Black/African American women menthol smokers who live in rural areas with low resources, like Phillips and Lee counties in Arkansas, will likely need additional support to completely quit all tobacco products.

Smokers in the present study were recruited from rural counties in Arkansas with poor resources and sparse access to health care services [28]. Such socioeconomic deprivation and limited resources can influence smokers’ access to evidence-based treatment programs, including provider advice to quit and individual and group counseling. Although we did not report specific methods that smokers used in the past year to quit smoking due to insufficient sample size, fewer than five participants reported using evidence-based pharmacotherapies (i.e., nicotine replacement therapy, bupropion, and varenicline), state quit line/telephone helpline services, one-on-one counseling, or a smoking cessation clinic/class/support group. Findings from the present study highlight the critical need to increase funding, awareness, and access to smoking cessation services, behavioral counseling, and evidence-based pharmacotherapies in Phillips and Lee counties in Arkansas and other low-resource, rural communities throughout Arkansas and similar Southern U.S. or Tobacco Nation communities. Improved access to such smoking cessation services could help to increase quitting success among socioeconomically deprived Black/African American women menthol smokers. Future research is also needed to determine the types of interventions that are best suited for socioeconomically deprived Black/African American women smokers in Arkansas to help reduce their nicotine dependence and menthol smoking.

### Strengths and Limitations

Baseline data from this study were collected from Black/African American women caregiver smokers living in low-resource counties in rural Arkansas, a Tobacco Nation state. The present study recruited smokers using homogenous convenience sampling and peer referrals, which are common methods used in community-based studies of underrepresented and hard-to-reach groups, where a list of persons eligible in community settings are not available [32,34,35]. While there are disadvantages related to the generalizability/sample estimates of non-probability sampling methods compared to probability sampling methods, our use of non-probability sampling methods was acceptable. Lee and Philips counties are rural counties located in the Arkansas Delta, and their geographic and social environment [28] would make it extremely challenging, as well as time and cost-intensive, to employ probability sampling (i.e., access and randomly sample the target population).

However, our strategic use of homogenous convenience sampling/narrow inclusion criteria was appropriate given the purpose of the study and allowed for a clearer, albeit narrower, generalizability of our findings [32,33]. For example, Black/African American women who reside in Lee and Phillips county, Arkansas have consistent patterns of tobacco use and similar social and socioeconomic backgrounds [28]. Thus, while the findings from the present study are not generalizable to the greater U.S. population of Black/African American women smokers, our findings are likely generalizable to Black/African American women caregiver smokers from our intervention counties and may extend more broadly to Black/African American women caregivers who smoke and reside in rural and/or low-resource communities in the U.S. South or Tobacco Nation States.

Although not reported in this study, smoking status was verified using expired carbon monoxide; however, we were not able to validate household smoking using saliva samples due to the onset of the COVID-19 pandemic. Despite the small sample size, the present study provides novel data, as few studies have reported any data on smoking, and menthol smoking in particular, social deprivation, nicotine dependence, and quitting behaviors among rural Black/African American women who face many social structural barriers to smoking cessation. These data are not generalizable, but our results are similar to what other studies have found among Black/African American women [14,17,51,52,53]. It is also plausible that our data may reflect what future studies could find among Black/African American women smokers who live in similar rural, low-resource counties in the South. Because women in our sample were severely socioeconomically disadvantaged, it was difficult to identify variations in socioeconomic deprivation, as most women had incomes of less than $10,000 per year. Studies with more rigorous methodology and larger, more diverse samples of Black/African American women from different socioeconomic backgrounds will help to clarify the relationships between menthol smoking status, socioeconomic deprivation and the outcomes of nicotine dependence and quitting behaviors among Black/African American women smokers overall.

## 5. Conclusions

Combustible tobacco use is still an epidemic among Black/African American women in Arkansas and who face severe socioeconomic deprivation. Such socioeconomic inequities contribute to the long-standing disparities observed among this understudied group. The present study shows that menthol smoking is associated with greater nicotine dependence among Black/African American women smokers who live in low-resource, rural counties. Our findings are similar to what other studies have found and further demonstrate that socioeconomic deprivation may increase the abuse liability of cigarettes among women from this background. As the FDA prepares to ban menthol in cigarettes and all characterizing flavors in cigars in the U.S., additional support is still needed to reduce the burden of tobacco among Black/African American women smokers in Phillips and Lee counties in Arkansas and similar communities the U.S. South. Moreover, increased access to services that support smoking cessation and additional research is needed to identify strategies to decrease nicotine dependence and improve successful quitting among Black/African American menthol smokers, especially those who are severely socioeconomically deprived and/or geographically isolated in Tobacco Nation States.

## Figures and Tables

**Table 1:** Sociodemographic Characteristics of Black/African American Women Smokers by Menthol Smoking Status (N = 146).

Sociodemographic Characteristics	Overall Sample % (*n*) or M (SD)	Current Menthol Smoking Status (Cigarette or LCC) % (*n*) or M (SD)	
		Any Menthol	Non-menthol
		69.2 (101)	30.8 (45)
			
Age	33.3 (8.7)	34.3 (8.1)	31.2 (9.7)
Race			
Black/African American only	94.4 (136)	94.0 (94)	95.5 (42)
Biracial/Multiracial	5.6 (8)	6.0 (6)	4.6 (2)
Sexual Orientation			
Heterosexual/Straight	73.6 (106)	71.7 (71)	77.8 (35)
Gay/Lesbian/Bisexual/Other	26.4 (38)	28.3 (28)	22.2 (10)
Body Mass Index (BMI)	32.2 (9.6)	31.9 (10.3)	32.7 (7.7)
Marital Status			
Married	25.0 (36)	29.3 (29)	15.6 (7)
Living with partner	15.3 (22)	17.2 (17)	11.1 (5)
Other	15.3 (22)	15.2 (15)	15.6 (7)
Never married	44.4 (64)	38.4 (38)	57.8 (26)
Employment Status			
Full-time	20.7 (30)	19.0 (19)	24.4 (11)
Part-time	16.6 (24)	13.0 (13)	24.4 (11)
Do not currently work for pay	62.8 (91)	68.0 (68)	51.1 (23)
Insurance Status			
Insured	81.1 (116)	78.6 (77)	86.7 (39)
Uninsured	18.9 (27)	21.4 (21)	13.3 (6)
Enrolled in Degree Program			
Yes	15.9 (23)	13.0 (13)	22.2 (10)
No	84.1 (122)	87.0 (87)	77.8 (35)
Education *			
Less than high school	26.9 (39)	31.0 (31)	17.8 (8)
High school or GED	48.3 (70)	51.0 (51)	42.2 (19)
Some college or associates degree	24.8 (36)	18.0 (18)	40.0 (18)
Annual Household Income			
<$10 K	65.5 (95)	64.0 (64)	68.9 (31)
≥$10 K	34.5 (50)	36.0 (36)	31.1 (14)
Received SNAP Benefits in Past Year			
Yes	81.4 (118)	84.0 (84)	75.6 (34)
No	18.6 (27)	16.0 (16)	24.4 (11)

M, mean; SD, standard deviation; LCC, little cigar/cigarillo; SNAP, Supplemental Nutrition Assistance Program. BMI of ≥30 indicates obesity, and participants who identified as Biracial/Multiracial reported Black/African American race as part of their racial background. Row sample sizes may not add up to the total sample (*n* = 146) due to missingness. * *p* < 0.05.

**Table 2:** Smoker and Other Substance Use Characteristics by Menthol Smoking Status (*n* = 146).

Tobacco/Other Substance Use Characteristic	Overall Sample % (*n*) or M (SD)	Any Current Menthol Smoking % (*n*) or M (SD)	
		Menthol 69.2 (101)	Non-Menthol 30.8 (45)
Age First Smoked Cigarettes Fairly Regularly	18.6 (5.0)	18.9 (5.2)	17.2 (4.1)
Age First Smoked LCCs Fairly Regularly	21.1 (7.4)	21.6 (7.5)	20.7 (7.4)
Daily Cigarette Smoking (Yes) ***	61.3 (92)	81.0 (81)	24.4 (11)
Daily LCC Smoking (Yes) ***	15.3 (23)	7.0 (7)	35.6 (16)
Number of Days Smoked Cigarettes in Past 30 Days ***	20.2 (10.5)	21.7 (10.2)	12.9 (9.2)
Cigarettes Smoked Per Day (Median (IQR)) **	8.0 (10.0)	10.0 (15.0)	5.0 (6.0)
Number of Days Smoked LCCs In Past 30 Days **	12.4 (11.2)	8.6 (9.6)	16.1 (11.4)
LCCs Smoked Per Day	6.4 (6.5)	5.8 (6.1)	6.9 (7.0)
Past 30-day Brand of LCC Smoked			
Black & Mild	61.0 (36)	58.6 (17)	63.3 (19)
Swisher Sweets	28.8 (17)	34.5 (10)	23.3 (7)
Other	10.2 (6)	6.9 (2)	13.3 (4)
Current Other Tobacco Product Use ** (Yes)	29.7 (43)	22.0 (22)	46.7 (21)
Current Alcohol Use (Yes)	37.2 (54)	34.0 (34)	44.4 (20)
Current Marijuana Use (Yes)	26.4 (38)	22.0 (22)	36.4 (16)
Current Blunt (Yes)	50.6 (40)	47.7 (21)	54.3 (19)
Current Other Drug Use (Yes)	2.8 (4)	2.0 (2)	4.4 (2)
Action Taken if Menthol Cigarettes Were Banned			
Switch to non-menthol cigarette	18.1 (21)	18.2 (18)	17.7 (3)
Switch to LCC	22.4 (26)	20.2 (20)	35.3 (6)
Quit any cigarette smoking	25.0 (29)	25.3 (25)	23.5 (4)
Switch to marijuana	11.2 (13)	12.1 (12)	5.9 (1)
None of the above	23.3 (27)	24.2 (24)	17.7 (3)
Nicotine Dependence and Quitting Behaviors			
FTND Score *	4.5 (2.2)	4.7 (2.0)	3.4 (2.6)
Time to First Cigarette of within 5 min **	50.0 (60)	55.6 (55)	23.8 (5)
Time to First LCC of within 5 min	32.8 (19)	39.3 (11)	26.7 (8)
Time to First Tobacco Product Use of within 5 min ***	48.0 (70)	57.4 (58)	36.7 (12)
Ever Tried to Quit Cigarettes	54.2 (64)	50.5 (49)	71.4 (15)
Tried to Quit Cigarettes in Past 12 Months (yes)	56.5 (35)	56.3 (27)	57.1 (8)
Planning to Quit Cigarette Smoking in Next 30 Days (yes)	19.8 (23)	18.8 (18)	25.0 (5)

M, mean; SD, standard deviation; LCC, little cigar/cigarillo; FTND, Fagerstrom test for nicotine dependence; Min, minutes. Cigarette/LCC related measures were assessed among all current cigarette/LCC smokers (i.e., among exclusive cigarette/LCC users and dual users, respectively). Past year cigarette quit attempts were only assessed among those who had ever tried to quit smoking cigarettes. Row sample sizes may not add up to the total sample (*n* = 146) or sub-sample sizes (*n* = 120 or *n* = 59) due to missingness. * *p* < 0.05, ** *p* < 0.01, *** *p* < 0.001.

**Table 3:** Adjusted Odds of Nicotine Dependence and Ever Engaging in Cigarette Quit Attempts.

	Outcomes		
Predictor	Time to First Tobacco Product Use (≤5 min vs. >5 min) AOR (95% CI)	FTND Score B (SE), t, *p*	Ever Tried to Quit Cigarettes (Yes vs. No) AOR (95% CI)
**MODEL 1**			
Any menthol product use (yes vs. no)	**4.10 (1.73, 9.70)**	-	0.36 (0.11, 1.12)
Menthol cigarette use (yes vs. no)	-	**1.49 (0.56) t = 2.68, *p* = 0.0085**	-
**MODEL 2**			
Any menthol product use (yes vs. no)	**5.90 (1.16, 22.33)**		0.45 (0.12, 1.72)
Menthol cigarette use (yes vs. no)	-	**1.16 (0.58), t = 2.01, *p* = 0.0469**	-

AOR, adjusted odds ratio; CI, confidence interval; FTND, Fagerstrom test for nicotine dependence. Boldface indicates statistical significance at the α = 0.05 level. Model 1: Analyses adjusted for age, education, income, sexual orientation, and marital status (dichotomized as married vs. all else/not married in all regression analyses). Model 2: Analyses predicting time to first tobacco product use were adjusted for age, education, income, sexual orientation, marital status, other tobacco product use, log transformed number of cigarettes smoked per day, and the number of days participants smoked cigarettes in the past 30 days. Analyses predicting FTND scores were adjusted for age, education, income, sexual orientation, marital status, other tobacco product use, and the number of days participants smoked cigarettes in the past 30 days.

**Table 4:** Depth of Socioeconomic Deprivation by Time to First Tobacco Product Use, Time to First Cigarette Use, and FTND Score.

	Overall N = 146	Low Education	Low Education and Low Income	Low Education, Low Income, and SNAP Recipient
	Cigarette and/or LCC smokers			
	*n* = 146	*n* = 39	*n* = 32	*n* = 23
	% (*n*)	% (*n*)	% (*n*)	% (*n*)
Time to First Tobacco Product Use ≤5 min of waking (Yes)	47.9 (70)	59.0 (23)	62.5 (20)	65.2 (15)
	**Cigarette smokers**			
	*n* = 120	*n* = 37	*n* = 31	*n* = 22
	% (*n*) or M (SD)	% (*n*) or M (SD)	% (*n*) or M (SD)	% (*n*) or M (SD)
Time to First Cigarette Use ≤5 min of waking (Yes)	50.0 (60)	59.5 (22)	61.3 (19)	63.6 (14)
FTND Score	4.6 (2.3)	4.9 (2.5)	4.9 (2.6)	4.9 (2.8)

SNAP, Supplemental Nutrition Assistance Program; LCC, little cigar/cigarillo; M, mean; SD, standard deviation; FTND, Fagerstrom test for nicotine dependence; Min, minutes. Low education was classified as a ‘12th grade education, no diploma’ or lower, low income was classified as an annual household income of <$10,000, and SNAP recipients received SNAP benefits in the past 12 months.

## Data Availability

The data presented in this study are available on request from the corresponding author. The data are not publicly available due to on-going data collection.
